# Prevalence and Burden of Cancer in Wolaita Zone, Ethiopia: A Retrospective Pathology‐Based Analysis

**DOI:** 10.1155/bmri/9923869

**Published:** 2025-12-17

**Authors:** Desta Seba Burka, Tamrat Balcha Balla, Temesgen Tesfaye Ajabo

**Affiliations:** ^1^ Addis Tena Primary Hospital, Wolaita Sodo, Ethiopia; ^2^ School of Pharmacy, College of Health Sciences and Medicine, Wolaita Sodo University, Wolaita Sodo, Ethiopia, wsu.edu.et; ^3^ School of Medicine, College of Health Sciences and Medicine, Wolaita Sodo University, Wolaita Sodo, Ethiopia, wsu.edu.et

**Keywords:** biopsy, cancer incidence, fine needle aspiration

## Abstract

**Background:**

Despite the increasing incidence of cancer worldwide, the knowledge about the trend of cancer incidence in Ethiopia is limited. The paucity of core cancer diagnostic services like pathology, diagnostic imaging technology, and the absence of a comprehensive national cancer registry masked the exact magnitude of cancer incidence in Ethiopia in general and the Wolaita area in particular. This study is aimed at filling the gap by analyzing diagnostic data from a referral clinic. The clinic used to serve as a primary diagnostic center for patients referred from over 25 healthcare facilities in the region.

**Methods:**

A pathology sample retrospective analysis–based prevalence study was conducted for the period between December 2017 and February 2022. Records saved in computers were subjected to analysis by using Statistical Package for Social Sciences (SPSS) software Version 22. The data were used to analyze the types and distribution of cancers in the region across age, sex, and diagnosis.

**Results and Discussions:**

The results showed notable gender disparities, with women experiencing a greater prevalence of breast cancer and men mostly receiving diagnoses for soft tissue sarcomas. The most prevalent forms of cancer were determined, along with the locations of each. The study also emphasized how different referral facilities, such as general hospitals, primary hospitals, and medium‐sized clinics, had varying cancer incidence rates. Although generalizability may be limited by the study′s clinic‐based design, its relevance to comparable healthcare settings in Ethiopia and other low‐resource locations is strengthened by the large and diverse sample drawn from a variety of referral institutions. This study emphasizes the necessity of focused screening programs and greater cancer awareness in Wolaita Zone, particularly in rural regions. The results also suggest possible directions for future investigation, such as population‐based studies to confirm and build upon these findings.

**Conclusions:**

This study provides crucial insights into the cancer burden in Wolaita Zone and emphasizes the importance of improving diagnostic and preventive measures. Further research, including broader, population‐based studies, is necessary to confirm these findings and inform regional cancer control strategies.

## 1. Introduction

Noncommunicable diseases including cancer are the leading causes of death worldwide. Cancer cells are abnormal cells that grow out of control. They spread beyond their site of origin and invade adjoining parts of the body or distant organs [1]. According to the World Cancer Report of the World Health Organization (WHO) for 2016, an estimated 70% of premature deaths were attributable to noncommunicable diseases of which more than 80% occurred in low‐ and medium‐income countries [2]. Most health systems in these countries including Ethiopia give major attention to the control of communicable diseases such as human immunodeficiency virus/acquired immunodeficiency syndrome (HIV/AIDS), tuberculosis, malaria, and most recently COVID‐19. However, according to the World Cancer Report (2014), the mortality from cancer alone is greater than all these communicable diseases combined [3]. This escalation of the cancer burden in low‐ and middle‐income countries is partly due to increased longevity and changing lifestyle practices [4].

In 2022, there were 20 million new cancer cases, up from 19.3 million new cancer cases in 2020. There were 10.0 million cancer deaths worldwide which showed significant growth compared to 14.1 million and 8.2 million that occurred in 2012, respectively [5–7].

By the year 2040, this number is projected to increase to 28.4 million corresponding to a 47% increase in 2020 showing the incidence rate of cancer is increasing globally [8]. The most commonly diagnosed cancers were female breast cancer (11.7%), followed by lung cancer (11.4%), colorectal cancer (10.0%), prostate cancer (7.3%), and stomach cancer (5.6%). Lung cancer is the leading cause of cancer death (18.0% of the total cancer deaths), followed by colorectal (9.4%), liver (8.3%), stomach (7.7%), and female breast (6.9%) cancers [8].

Africa is a continent facing challenges from communicable diseases and the unprecedented growth in cancer burden at the same time. Cancer incidence is rising in parallel to the global cancer rate. It has increased from 715,000 in 2008 to 1.1 million in 2020, and the cancer mortality count has increased from 542,000 in 2008 to 711,000 in 2020 [9]. In 2022, there were 20 million new cancer cases and almost 10 million mortalities. Eastern Africa is one of the global regions where the risk of dying from cancer among women in 2022 was highest (10.7%) [6, 7].

Breast, cervical, prostate, liver, and colorectal cancer were the leading types of cancer accounting for 48% of new cases in Africa [6, 7, 10]. Between the year 2020 and 2040, the burden of cancer incidence is expected to increase the most in Africa compared to the rest of the world, rising from 1.1 million cases in 2020 to 2.1 million cases in 2040. This estimation can be even higher due to the escalation of behavioral and environmental risk factors, increasing life spans, and improvements in cancer registration [10].

According to the WHO report, in sub‐Saharan Africa a total of 801,392 new cancer cases and 520,158 cancer deaths were estimated to have occurred in 2020. The most common cancers among women are breast cancer (number one in 28 countries) and lung cancer (number one in 19 countries). In men, the most common cancer type was prostate cancer (77,300 cases), followed by liver cancer (24,700 cases) and colorectal cancer (23,400 cases). Prostate cancer was the leading incidence of cancer in men in 40 countries in sub‐Saharan Africa [11]. The region is predicted to have greater than 85% increases in cancer burden by the year 2030 [12].

Similarly, cancer incidence and mortality in Ethiopia are also increasing. Cancer incidence, death, and the disability‐adjusted life year (DALY) count increased by 32%, 29%, and 19% in the years 2010–2019 [13]. Although population‐based data are not available in this country except outside of Addis Ababa, the annual incidence of cancer is estimated to be 60,960 cases [14]. The most prevalent cancers in Ethiopia among the adult population are breast cancer (30.2%), cancer of the cervix (13.4%), and colorectal cancer (5.7%) [15].

Despite rising morbidity and mortality in Africa, cancer remains under represented in the research field and healthcare services. The major reasons for this include limited resources and other pressing public health problems including communicable diseases [16]. Despite the increasing burden of cancer, evidence about the pattern and burden of cancer in Ethiopia is inadequate in the literature. The scarcity of core cancer diagnostic services like pathology and diagnostic imaging technology and the absence of a comprehensive national cancer registry masked the exact magnitude of cancer in Ethiopia in general and the Wolaita area in particular.

Although there is a prompt report of the pattern of cancer in the Wolaita Sodo University Hospital revealing that the majority of the incidence occurred in females of the reproductive age group, there is still a huge gap in knowledge regarding the burden of cancer based on comprehensive clinical data using the institutional registry [17]. Thus, this study was aimed at assessing the pattern and burden of cancer incidence using an institution‐based registry through the years in the Wolaita zone.

## 2. Materials and Methods

### 2.1. Materials

We used Wright stain, Giemsa stain 0.76% *w*/*v* solution, alcohol, 10% formalin, normal saline, gauze, and cotton tip applicator, which were manufactured by Ethiopian Pharmaceuticals Manufacturing Sh.co Addis Ababa, Ethiopia; 21‐mm gauged syringe of 5 mL or 10 mL, frosted slide, lancet, clean gloves, surgical blade, spatula, and cover slide manufactured by Hangzhou and Changzhou Medical Devices Co. Ltd (China); microscope product of (Zeiss, Primo star, Germany), microtome (5 mm), tissue processor, cassettes, and litmus paper (Leica, Germany); ethanol, xylene, paraffin, hematoxylin‐eosin dye, dipex, and hydrochloric acid; and autoclave, centrifuge machine, water bath, embedding station, cooling plate, oven, bone marrow biopsy needle, surgical forces (toothed), speculum, tenaculum forceps, and knives of different sizes (GST Corporation Ltd, India) were used.

### 2.2. Methods

#### 2.2.1. Study Settings and Design

This study was conducted in the Wolaita Zone population. According to the 2020 population projection conducted by the Central Statistical Agency of Ethiopia (CSA), Wolaita Zone has a total population of 7,385,782. Although the inhabitants of the zone are served by more than 30 different healthcare facilities including one comprehensive specialized public hospital, two general nongovernmental hospitals, eight primary public hospitals, four primary private hospitals, and more than 20 medium clinics, none of them except for Addis Tena Medium Clinic used to render significant pathological diagnostic tests before June 2021. Addis Tena Clinic, a private clinic 330 km away from Addis Ababa located in Wolaita Sodo, is the capital of the South Ethiopia Region. The clinic renders general primary outpatient care and basic radiological and pathological diagnostic services for cancer suspects. Retrospective analysis of pathology results of FNAC, hematopathology, Papanicolaou (Pap) smear and BIOPSY were collected from Addis Tena Clinic from Dec 2, 2017 to Feb 28, 2022. Addis Tena Clinic was the only health facility rendering pathology services for all of Wolaita Zone from the year 2012 to June 2021. The study population comprised all encounters who visited the clinic for tumor investigations.

#### 2.2.2. Inclusion and Exclusion Criteria

All patients who visited the clinic during the period Dec 2, 2017 to Feb 28, 2022 for tumor investigation evidenced with registration of age, sex, and diagnosis were included in the study. However, clients with insufficient data regarding age, sex, and/or diagnosis were excluded.

#### 2.2.3. Diagnostic Procedures and Sample Processing

FNAC was performed using 5–10‐mL syringes, with aspirates evaluated for adequacy and cellular content, then smeared on slides and stained with Wright or Giemsa for cytological examination. Procedures followed standard techniques as outlined by Nayak [18]. Tissue specimens were processed through formalin fixation, dehydration, paraffin embedding, sectioning (6–8 *μ*m), and hematoxylin–eosin staining based on protocols from Marchand et al. [19]. Pap smear samples were collected using speculum and spatula/cytobrush methods and stained similarly, following Löffler and Rastetter [20]. Peripheral blood smears were prepared from fingertip pricks, stained with Wright stain, and examined for morphological evaluation. Bone marrow aspiration was performed using standard biopsy techniques and processed according to Azage et al. [21].

#### 2.2.4. Data Quality Assurance

Most of the FNAC and histopathology samples were referred from private and government hospitals and private clinics. There is a dedicated procedure room equipped with setups complying with the FNAC and biopsy quality requirements as per [18–21]. The quality of samples was reassured starting from the origin of the referring clinic and hospital where all the biopsy samples were preserved in 10% formalin with all the required clinical data on the referral slip filled. The biopsy samples were kept in isolated bits rooms with all required equipment. Smaller samples were awaited for a few days to 1 week for fixation and a bit longer time for bigger tissues. All samples were collected and reported by the pathologist. The tissue processing was performed by trained histopathology technologists.

#### 2.2.5. Inclusion Criteria

Patient records were included in the analysis only if they contained complete and accurate information on key variables such as age, sex, and confirmed diagnosis. Incomplete records with missing or ambiguous data were excluded to ensure the integrity and reliability of the study results. While this approach minimizes errors and misclassification in the analysis, it may introduce a selection bias by excluding cases that could represent a broader spectrum of the population.

#### 2.2.6. Study Limitation and Generalizability

The study is based on diagnostic data from a single clinic which may raise a question of generalizability with accurate representativeness especially since there might be those who are diagnosed in primary healthcare units or the others who do not seek diagnostic efforts. Moreover, the partial records which are necessary for accuracy were excluded for their incompleteness leaving subpopulations who have been misdiagnosed or not diagnosed left out at all.

However, that several medical facilities in Wolaita Zone and surrounding areas were involved through a referral network and because of this the study′s relevance and integrity are maintained by the large and diverse patients from 25 medical facilities whose names were anonymized to ensure anonymity and presented aggregated integrating ethical compliance and diversity of the referral facilities (Table 1).

**Table 1:** Referral sources for FNAC and biopsy specimen from health facilities in Wolaita Zone and Beyond (2017–2022).

**Health facility type**	**FNAC count (no.)**	**FNAC percentage (%)**	**Biopsy count (no.)**	**Biopsy percentage (%)**
General hospitals	7674	90.6	1864	89.6
Primary hospitals	464	5.48	115	5.53
Medium clinics	288	3.4	96	4.6
Not mentioned	40	0.48	5	0.24
Total	8466	100%	2080	100%

#### 2.2.7. Data Collection and Analysis

Data retrieval and cleaning were performed from the records in the personal computer of dedicated pathology and analyzed. The descriptive methods including the use of tables, figures, and summary statistics such as frequency distribution, mean, and standard deviation were computed with SPSS Version 23 and Microsoft Excel 2013 Software.

#### 2.2.8. Ethical Justification

This study used anonymized diagnostic data from healthcare facilities in Wolaita Zone. Consent for the use of data was obtained from Addis Tena Clinic, patient and institutional identifiers were removed, and data was aggregated to ensure confidentiality. Only nonidentifiable data (age, sex, and diagnosis) were included, and incomplete records were excluded to maintain accuracy. While IRB approval was not sought, the study adhered to ethical guidelines, ensuring participant privacy.

## 3. Results

### 3.1. Demographic Profiles and Rates of Detection of Malignancy

Despite a total of 11,300 clients with suspected malignancies being evaluated between December 2, 2017 and February 28, 2022, 1865 cases (16.5%) were confirmed to have cancer. All the rest showed benign conditions, inconclusive results, and nonmalignant findings not fulfilling the cancer diagnosis. Their demographic distribution including gender and age groups was described in Table 2. In addition, it describes the frequency distribution of each type of diagnostic method, the year the client visited, and the rate of malignancy detection among the total clients in each group across the independent variables.

**Table 2:** The demographic, method of diagnosis, and year of visit frequency distribution of rate of detection of cancer among the suspects visited Addis Tena Clinic from December 2017 to February 2022.

**Variables**	**Levels**	**No. of suspects. (%)**	**No. of confirmed malignancy (%)**
Gender	M	4147 (36.7)	727 (17.5)
	F	7153 (63.3)	1138 (15.9)

Age group (years)	0–14	1054 (9.3)	115 (10.9)
	15–24	2269 (20.1)	168 (7.4)
	25–54	6955 (61.5)	1209 (17.3)
	55–64	670 (5.9)	245 (36.6)
	≥ 64	352 (3.1)	128 (36.4)

Diagnostic procedures	FNAC	8466 (74.9)	1105 (13)
	Biopsy	2080 (18.4)	665 (32)
	Hematopathology	754 (6.7)	95 (12.6)

Year of visit diagnosis	2017	2838 (25.1)	500 (17.6)
	2018	2994 (26.5)	427 (14.3)
	2019	2148 (19.0)	297 (13.8)
	2020	1535 (13.6)	299 (19.5)
	2021	1784 (15.8)	342 (19.2)

### 3.2. The Incidence of Cancer Across Years

**Figure 1:**
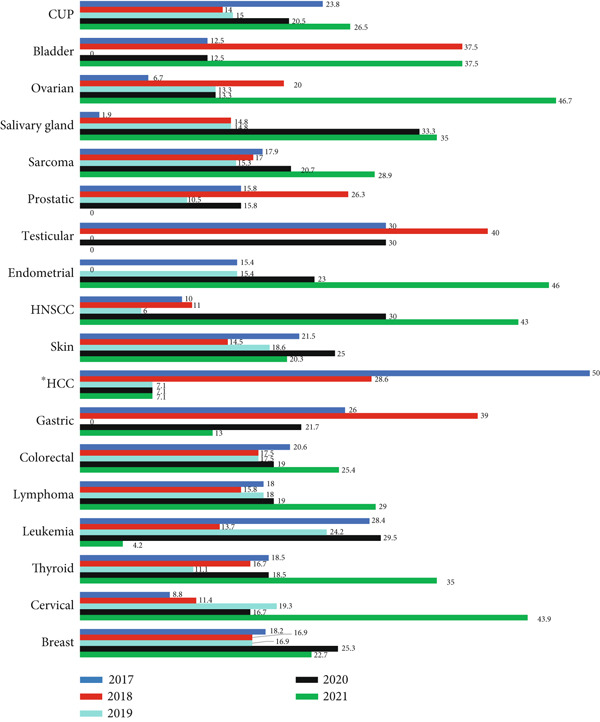
The number of each type of cancer in terms of frequency distribution seen in Addis Tena Clinic from December 2017 to February 2022 ( ^∗^HCC stands for hepatocellular carcinoma).

The burden of each type of cancer in terms of frequency distribution was described in Figure 1.

Similarly, gender‐specific burdens of each type of cancer from the year 2017–2022 and the relative incidence and prevalence in males and females in each of the types of cancers in the years 2017 through 2021 were compared in Tables 3 and 4, respectively.

**Table 3:** Annual distribution of the five most common cancers diagnosed among male clients at Addis Tena Clinic (2017–2022). Total confirmed male cancer cases = 727.

	**Cancer type**	**2017**	**2018**	**2019**	**2020**	**2021**	**5 years**
1	Sarcoma	39 (5.4%)	27 (3.7%)	21 (2.9%)	22 (3.0%)	23 (3.2%)	132 (18.2%)
2	Lymphoma	30 (4.1%)	26 (3.6%)	23 (3.2%)	17 (2.3%)	23 (3.2%)	119 (16.4%)
3	Skin cancer	19 (2.6%)	24 (3.3%)	17 (2.3%)	11 (1.5%)	22 (3.0%)	93 (12.8%)
4	^∗^HNSCC	27 (3.7%)	17 (2.3%)	2 (0.3%)	5 (0.7%)	6 (0.8%)	57 (7.8%)
5	Leukemia	1 (0.1%)	10 (1.4%)	17 (2.3%)	8 (1.1%)	19 (2.6%)	55 (7.6%)
6	Breast cancer	12 (1.6%)	7 (1.0%)	13 (1.8%)	4 (0.6%)	9 (1.2%)	45 (6.2%)
7	Colorectal cancer	11 (1.5%)	5 (0.7%)	7 (1.0%)	6 (0.8%)	7 (1.0%)	36 (5.0%)
8	Salivary gland tumor	6 (0.8%)	11 (1.5%)	7 (1.0%)	4 (0.6%)	1 (0.1%)	29 (4.0%)
9	Prostatic cancer	6 (0.8%)	3 (0.4%)	2 (0.3%)	5 (0.7%)	3 (0.4%)	19 (2.6%)
10	Thyroid cancer	4 (0.6%)	1 (0.1%)	3 (0.4%)	3 (0.4%)	4 (0.6%)	15 (2.1%)

^∗^HNSCC stands for head and neck squamous cell carcinoma.

**Table 4:** Annual distribution of the five most common cancers diagnosed among female clients at Addis Tena Clinic (2017–2022). Total confirmed female cancer cases = 1138.

	**Cancer type**	**2017**	**2018**	**2019**	**2020**	**2021**	**Total (5 years)**
1	Breast cancer	93 (8.2%)	110 (9.7%)	65 (5.7%)	74 (6.5%)	75 (6.6%)	417 (36.6%)
2	Cervical cancer	50 (4.4%)	19 (1.7%)	22 (1.9%)	13 (1.1%)	10 (0.9%)	114 (10.0%)
3	Sarcoma	42 (3.7%)	31 (2.7%)	22 (1.9%)	26 (2.3%)	27 (2.4%)	148 (13.0%)
4	Skin cancer	16 (1.4%)	19 (1.7%)	15 (1.3%)	14 (1.2%)	15 (1.3%)	79 (6.9%)
5	Lymphoma	23 (2.0%)	9 (0.8%)	10 (0.9%)	12 (1.1%)	10 (0.9%)	64 (5.6%)
6	Thyroid cancer	15 (1.3%)	9 (0.8%)	3 (0.3%)	6 (0.5%)	6 (0.5%)	39 (3.4%)
7	HNSCC	16 (1.4%)	13 (1.1%)	4 (0.3%)	6 (0.5%)	4 (0.3%)	43 (3.8%)
8	Leukemia	3 (0.3%)	18 (1.6%)	6 (0.5%)	5 (0.4%)	8 (0.7%)	40 (3.5%)
9	Colorectal cancer	5 (0.4%)	7 (0.6%)	4 (0.3%)	5 (0.4%)	6 (0.5%)	27 (2.4%)
10	Ovarian cancer	7 (0.6%)	2 (0.2%)	2 (0.2%)	3 (0.3%)	1 (0.1%)	15 (1.3%)

**Figure 2:**
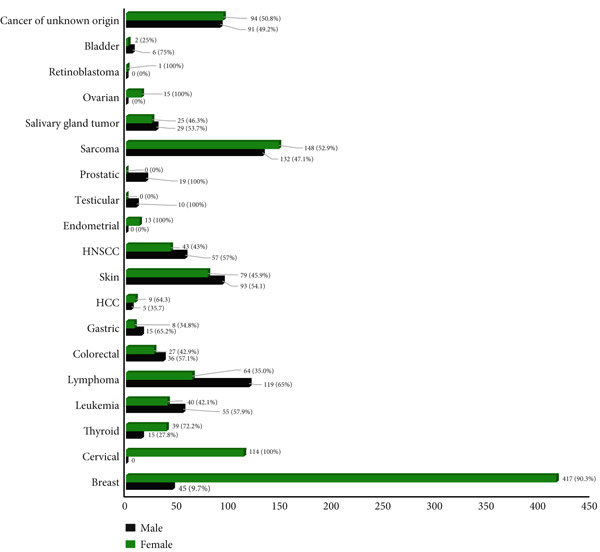
The frequency distribution of each type of cancer seen in Addis Tena Clinic from December 2017 to February 2022 in males versus females.

Figure 2 describes the comparative prevalence of each type of malignancy across gender variation.

Breast cancer is the most common cancer in patients above the age of 14 which accounts for (*n* = 462, 24.8%) followed by sarcoma, cancer of unknown primary, lymphoma, skin, and cervical cancers. In children under the age of 14, the most common cancers are lymphoma (*n* = 40, 35%), sarcoma (*n* = 34, 29.4%), and leukemia (*n* = 28, 24.5%). Table 5 describes the age group‐specific relative frequency distribution of each type of cancer seen in Addis Tena Clinic from December 2017 to February 2022.

**Table 5:** The frequency distribution of cancer types seen in Addis Tena Clinic from December 2017 to February 2022 across age groups.

**Age group in years**	**0–14**	**15–24**	**25–54**	**55–64**	**≥ 65**
	**No (%)**	**No (%)**	**No (%)**	**No (%)**	**No (%)**
Breast	0 (0)	10 (2.1)	395 (85.5)	44 (9.5)	13 (2.8)
Cervical	0 (0)	1 (0.87)	87 (76.3)	20 (17.5)	6 (5.2)
Thyroid	1 (1.8)	1 (1.9)	39 (72.2)	11 (20.3)	2 (3.7)
Leukemia	28 (29.5)	16 (16.8)	40 (42)	7 (7.3)	4 (4.2)
Lymphoma	41 (22.4)	26 (14.2)	78 (42.6)	23 (12.6)	15 (8.2)
Colorectal	0 (0)	4 (6.3)	39 (61.9)	10 (15.9)	10 (15.9)
Gastric	0 (0)	1 (4.3)	16 (69.6)	3 (13)	3 (13)
HCC	0 (0)	1 (7.1)	7 (0.5)	5 (35.7)	1 (7.1)
Skin	1 (0.6)	2 (1.1)	115 (66.8)	40 (23.3)	14 (8.1)
HNSCC	1 (1)	11 (11)	70 (7)	11 (11)	7 (7)
Endometrial	0 (0)	1 (7.7)	11 (84.6)	1 (7.7)	0 (0)
Testicular	0 (0)	2 (20)	6 (60)	0 (0)	2 (20)
Prostatic	0 (0)	0 (0)	1 (5.2)	7 (36.8)	11 (57.9)
Sarcoma	34 (12)	67 (23.9)	148 (52.8)	21 (7.5)	10 (3.6)
Salivary gland tumor	0 (0)	9 (16.7)	33 (61.1)	7 (12.9)	5 (9.2)
Ovarian	0 (0)	0 (0)	11 (73.3)	2 (13.3)	2 (13.3)
Retinoblastoma	1 (100)	0 (0)	0 (0)	0 (0)	0 (0)
Bladder	0 (0)	0 (0)	2 (25)	2 (25)	4 (50)
CUP	8 (4.3)	16 (8.6)	111 (60)	31 (16.8)	19 (10)

In children of age less than 14, lymphoma (*n* = 41, 35.6%) is the most common cancer whereas breast cancer is the most common cancer in patients above the age of 14 which accounts for (*n* = 462, 24.8%).

**Figure 3:**
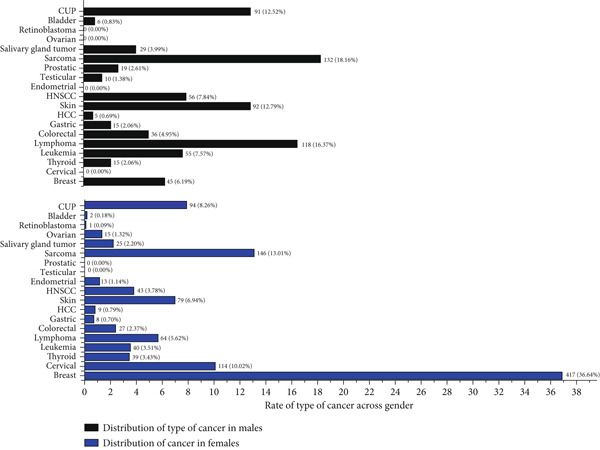
The comparative incidence and prevalence of each type of cancer in males versus females seen in Addis Tena Clinic from December 2017 to February 2022.

The gender‐specific comparative incidence of each type of cancer seen in Addis Tena Clinic from December 2017 to February 2022 was presented in Figure 3.

## 4. Discussion

Out of a total of 11,300 pathology samples investigated for malignancies, 1865 (16.5%) were diagnosed with confirmed malignancies between the years 2017 and 2022 (Table 2). The majority of patients with confirmed malignancy were females 1138 (61.0%) whereas males accounted for 727 (39.0%) which shows women are affected 22% more frequently than men (Figures 2 and 3) which is evidently due to the higher rates of breast and cervical cancer among women. A similar finding was seen at Felege Hiwot Referral Hospital, Northwest Ethiopia where more than two‐thirds of affected patients were females [22]. However, it contrasts with global cancer incidence where men were 19% more commonly affected than women [5].

Increasing age is one of the most important risk factors for cancer. The incidence of breast cancer increases steadily with age [23]. This similar pattern was also noticed in our study where patients in their 60s and above were the most commonly affected age groups (39.0%) whereas patients between 15 and 24 years of age were the least affected (7.4%). Although the trend of cancer incidence was erratic in the past 5 years, the overall cancer detection rate showed an increasing pattern. This coincided with the report on the national burden and trend of cancer in Ethiopia where age‐standardized cancer incidence increased by 5% from the years 2010 to 2019 [13]. The highest cancer detection rate was observed in the year 2020 (19.5%) and 2021 (19.2%) whereas a smaller number of patients were diagnosed in the year 2018 (14.3%) and 2019 (13.8%). Fine needle aspiration cytology was the major diagnostic procedure used (74.9% of cases) but the maximum number of cancer diagnoses (32%) was made through histopathology (biopsy); only 13% and 12.6% of cancer diagnoses were made by FNAC and hematopathology procedures, respectively. Although FNAC is an easy, cheap, comfortable, and safe procedure to perform, the sensitivity and specificity of histopathology are superior to FNAC. The sensitivity of histopathology is 100% compared to FNAC which is 95% sensitive with a false negativity rate of 5% [24].

As it is shown in Figure 1, the Top 5 leading cancers diagnosed throughout the year 2017–2022 were breast, soft tissue sarcoma, cancer of unknown primary, lymphoma, and skin cancer. Skin cancer outnumbered lymphoma in the year 2018 and 2021. The incidence of these cancers in Wolaita is partly different from the findings reported worldwide where breast, lung, and prostate are the most frequently diagnosed cancers [25]. These differences could possibly be explained by differences in the standard of living, exposure to risk factors like cigarette smoking, demographic factors like advanced aging, and barriers to high‐quality cancer prevention. Retinoblastoma of the eye was the least identified cancer throughout the years 2017–2022, along with testicular, liver, bladder, gastric, endometrial, and ovarian cancers appearing in different orders.

According to Tables 3 and 4, the most common cancers identified in females were breast, soft tissue sarcoma, cervical cancers, cancer of unknown primary, and skin cancers. The least frequently seen cancers include retinoblastoma, bladder, and liver cancer (except for the year 2021 where the highest number of liver cancer cases, six (66.7%) were identified). All types of cancer incidences showed an erratic trend. The highest number of breast cancer cases, 110 (26.4%) was diagnosed in the year 2018, 93 (22.3%) in 2017, 75 (18%) in 2021, and 74 (17.7%) in 2020 whereas the lowest number 65 (15.6%) was diagnosed in the year 2019. The most common cancers diagnosed in males were sarcoma and lymphoma whereas the least frequently seen cancers were liver, testicular, and bladder cancer. The highest number of soft tissue sarcoma cases, 39 (29.5%) was identified in the year 2017 whereas the least number 21 (15.9%) was diagnosed in the year 2019.

In females, breast cancer was the commonest cancer constituting about 36.6%, followed by soft tissue and bone (13%), cervical cancer (10%), and cancer of unknown primary (8.26%). Similar findings were also reported from a study done in Addis Ababa′s population‐based cancer registry and the Tigray region where breast cancer constituted about 33% and 32.3%, respectively [14, 26]. Although one hospital‐based study conducted at Tikur Anbessa Hospital in Addis Ababa, showed cervical cancer of the uterus is the most common cancer (39.7%), several reports confirm that breast cancer is the most common tumor in women and its incidence is increasing in other East African countries [27, 28]. Some risk factors for breast cancer are similar in Ethiopia and industrialized countries. The rising incidence of breast cancer may be attributed to increasing risk factors associated with globalization and a growing economy, which brought dramatic changes in lifestyle and an increase in the proportion of women in the industrial workforce. This resulted in a convergence toward the risk factor profile of Western countries (postponement of childbearing, having fewer children, and increased physical inactivity) narrowing international gaps in breast cancer morbidity [5].

As per cervical cancer, sexually transmitted HPV (human papilloma virus) infection is the culprit for its development. Known risk factors associated with HPV infection are early marriage, beginning sexual intercourse at an early age, previous STI, having multiple sexual partners, low socioeconomic status, and being HIV positive. Globally, the prevalence of infection with HPV in women without cervical abnormalities is 11%–12%, with higher rates in sub‐Saharan Africa (24%). This number is even higher in eastern Africa where 35.8% of women in the general population are estimated to harbor cervical HPV infection at a given time [29]. Cervical cancer is believed to be 100% preventable through control of risk factors, effective vaccination, implementation of screening techniques, and treating early precancerous lesions. However, the lack of effective population‐based level screening programs, very limited resources, and poor awareness about disease prevention are major reasons for the higher prevalence of cervical cancer in the region. Immediate treatment of precancerous lesions after screening with one‐time HPV testing or visual inspection with acetic acid (VIA), has the greatest impact and is the most cost‐effective cervical cancer prevention strategy [29]. Cancer of unknown primary is a heterogeneous group of malignant metastatic cancers in which thorough standardized diagnostic work‐ups failed to identify primary sites [30]. Our findings coincide with global reports where cancer of unknown primary is the sixth to eighth most common cancer accounting for 2.3%–5% among newly diagnosed cancers [31]. The observed late presentation, probably due to limited awareness and then access to pathological cancer diagnosis, and using only pathologic diagnostic work‐up might have resulted in a substantial number of cancer of unknown primary.

In males, the most common cancers were bone and soft tissue cancers (18.1%), followed by lymphoma (16.3%) skin cancers (12.7%), cancer of unknown primary (12.5%), and head and neck squamous cell carcinoma (7.8%). Soft tissue sarcomas are complicated malignancies encompassing at least 100 different histologic and molecular subtypes representing a cohort of rare and heterogeneous tumors that account for 1% of all adult malignancies [32, 33]. A similar finding was seen in a hospital‐based retrospective cross‐sectional study conducted at Tikur Anbessa Hospital and Wolaita Sodo University Hospital, where bone and soft tissue cancer was the most common finding in men [17, 25]. However, the result is different from global reports where in many countries lung cancer is the most frequent cancer in men followed by prostate and stomach cancer [6, 34]. This disparity could be due to the ease and accessibility of superficial sites for pathology sampling in comparison to deep and visceral cancers which require more advanced and invasive procedures that are less frequently performed in our study area.

Another striking finding of this study was male breast cancer. Its proportion was 9.7% of all breast cancer patients and 6.1% of all cancer incidence in men. This report is similar to a hospital‐based study conducted at Tikur Anbessa Hospital in Addis Ababa and neighboring country Kenya where male breast cancer incidence was about 5.5% and 7%, respectively [5, 25]. Another high proportion of male breast cancer is also noted in a cross‐sectional study done at the university hospital of Gonder in northern Ethiopia (18%) [35]. However, it contrasts with the finding from the United States of America where male breast cancer makes up less than 1% of all cancers in men and less than 1% of all breast cancers [36].

As it is described in Table 5, the most common childhood cancers under the age of 14 were lymphoma (35.6%), bone and soft tissue cancers (29.5%), and leukemia (24.3%) of all childhood cancers whereas another typical childhood tumors like retinoblastoma, nephroblastoma, neuroblastoma, and central nervous system (CNS) tumors were least or have never been identified in our study. Lymphoma and leukemia alone comprised 59.9% of childhood cancers. This is consistent with the study done in Gondar University Hospital, Northwest Ethiopia, where leukemia and lymphoma account for about 43% making it the most prevalent childhood cancers [37]. This finding was also in accordance with the study done in central Sudan where leukemia (29%) and lymphomas (26%) comprised 55% of all pediatric cancers [38]. However, it contrasts with reports from high‐income countries like the United States, where the most common childhood cancers were acute lymphoblastic leukemia (ALL) (26%), brain and CNS tumors (21%), and neuroblastoma (7%) [39]. The reason for this disparity could be ethnic variation and limited availability of neurosurgical procedures and diagnostic imaging in resource‐limited settings like our study area. However, the higher incidence of lymphoma and leukemia in our study is difficult to fully explain.

### 4.1. Limitations of the Study

Since this study involved data originally not intended for research and only those who could afford the diagnosis and access might have visited the clinic, the findings may not represent the general population. Also, age‐standardized incidence rates were not calculated, and thus, they may not accurately be used to determine population‐wide incidence, and the sarcoma subclassification was not performed.

## 5. Conclusions

In conclusion, the present study demonstrates that cancer is an increasingly serious health problem in Wolaita. The burden of cancer incidence was variable in the past 5 years. The majority of cases were females of reproductive age group. Preventable cancers with effective screening tests such as cervical and breast cancers were seen in a significant proportion. On the other hand, tumors of deep‐seated organs such as lung, brain, colon, and prostate were seen with lower incidence. Creating awareness and implementing preventive care along with effective educational and screening strategies is needed in the area to mitigate the growing incidence of malignancy. Comprehensive demographic and clinical data using population or facility‐based cancer registry is required to confirm and build on these findings and get better information for planning and monitoring cancer patterns in the region.

**Nomenclature**
ALL: acute lymphoblastic leukemia
FNAC: fine needle aspiration cytology
WHO: World Health Organization
PAP smear: Papanicolaou smear
CNS: central nervous system
EBV: Epstein–Bar virus
HIV: human immunodeficiency disease
HPV: human papilloma virus

## Ethics Statement

This research was solely based on highly anonymized pathology records with no personal identifiers, and thus, the institutional ethical approval was not required in accordance with the local and international guidelines.

## Conflicts of Interest

The authors declare no conflicts of interest.

## Funding

No funding was received for this manuscript.

## Data Availability

The relevant data is available from the corresponding author upon reasonable request.
